# Liquid Biopsies in the Early Diagnosis, Prognosis, and Tailored Treatment of Colorectal Cancer

**DOI:** 10.3390/cancers17060927

**Published:** 2025-03-08

**Authors:** Efstathia Liatsou, Ioannis Kollias, Maria Trapali, Diamantis I. Tsilimigras, Maria Gavriatopoulou, Ioannis Ntanasis-Stathopoulos

**Affiliations:** 1 CAST, Center of Allogenic Transplantation and Cell Therapies, Karolinska University, 17177 Stockholm, Sweden; fayliatsou1@gmail.com; 2 Department of Clinical Therapeutics, National and Kapodistrian University of Athens, 11528 Athens, Greece; ikollias@biol.uoa.gr (I.K.); mgavria@med.uoa.gr (M.G.); 3 Laboratory of Chemistry, Biochemistry and Cosmetic Science, Department of Biomedical Medicine, University of West Attica, 12243 Egaleo, Greece; ymaria@uniwa.gr; 4 Department of Surgery, Division of Surgical Oncology, The Ohio State University Wexner Medical Center and James Comprehensive Cancer Center, Columbus, OH 43210, USA; diamantis.tsilimigras@osumc.edu

**Keywords:** liquid biopsy, colorectal cancer, diagnosis, prognosis, treatment, circulating tumor cells, circulating tumor DNA, minimal residual disease, non-coding RNAs

## Abstract

Introduction: Liquid biopsies provide a less-invasive option to tissue biopsies for the early diagnosis, prognosis, and tailored therapy of colorectal cancer (CRC). CRC is a major cause of cancer-related death, and early identification is essential for improving patient outcomes. Review: Conventional diagnostic techniques, including colonoscopy and tissue biopsy, may be enhanced by liquid biopsies that examine circulating tumor cells (CTCs), circulating tumor DNA (ctDNA), extracellular vesicles (EVs), and other indicators present in body fluids. These markers provide significant insights into tumor biology, heterogeneity, and therapeutic response. CTCs detected in early-stage CRC have prognostic significance for disease recurrence and survival, while ctDNA investigation may uncover genetic mutations, epigenetic alterations, and tumor development. The identification of ctDNA in minimal residual disease (MRD) postsurgery correlates with an elevated risk of recurrence and unfavorable prognosis, underscoring its use in assessing treatment effectiveness. Furthermore, non-coding RNAs (ncRNAs) contained inside EVs provide potential prospective biomarkers and therapeutic targets, facilitating diagnosis and treatment assessment. Notwithstanding the potential of liquid biopsies, obstacles persist in assay standardization, sensitivity enhancement, and the management of tumor heterogeneity. Additional extensive research is required to determine their function in clinical practice. Conclusion: Overall, liquid biopsies serve as a potential instrument for real-time monitoring, evaluating therapy responses, and directing individualized therapeutic strategies in CRC patients.

## Simple Summary

Liquid biopsies are a less invasive alternative to traditional tissue biopsies. Liquid biopsies analyze markers in body fluids like circulating tumor cells (CTCs), circulating tumor DNA (ctDNA), and extracellular vesicles (EVs), which provide insights into tumor behavior and treatment response. CTCs in early-stage colorectal cancer (CRC) may predict disease recurrence and survival. Detecting ctDNA after surgery helps assess treatment effectiveness and recurrence risk. Non-coding RNAs (ncRNAs) in EVs also evaluate potential biomarkers and therapeutic targets. However, challenges remain in standardizing assays, enhancing sensitivity, and managing tumor heterogeneity. Further research is needed to confirm role of liquid biopsies in clinical practice.

## 1. Introduction

Colorectal cancer (CRC) is one of the most prevalent types of cancer, being third in incidence in both men and women, and it is ranked second in terms of mortality for men and women combined [1]. Early detection of the disease is crucial for the outcome of patients with CRC. It is shown that the survival rates for both early-stage colon cancer (CC) and rectal cancer (RC) are significantly higher than those for advanced stages of both subtypes [2].

Although colonoscopy and tissue biopsy remain the standard procedures for the detection and diagnosis of CRC, liquid biopsy, a broad set of minimally invasive tests on body fluids, gives a great amount of important complementary information about tumor biology and heterogeneity, helps with disease prognosis and monitoring, as well as aiding in early detection and tailored treatment strategies to manage the disease [3]. Another significant application of the detection of cancer-specific biomarkers by liquid biopsy methods is the longitudinal monitoring of patients during and after treatment as well as post-operatively with the aim of the early detection of minimal residual disease (MRD). The purpose of MRD monitoring is to gain a comprehensive view of the risk of relapse that could lead to the selection of patients that require treatment escalation [4].

New techniques for the enrichment and analysis of molecules and biomarkers such as circulating tumor cells (CTCs), circulating tumor DNA (ctDNA), tumor-educated platelets (TEPS), extracellular vesicles (EVs), and proteins in the peripheral blood or other body fluids of patients are rapidly evolving to aid in the early detection of the disease and tracking of the molecular evolution and epigenetic changes under active chemotherapy treatment. Disease progression is monitored in a less invasive and more precise way by implementing liquid biopsies in the follow-up strategy during systemic treatment. Moreover, by utilizing liquid biopsy approaches, the heterogeneous genetic identity of the growing tumor-cell clones is mapped, allowing practitioners to rechallenge ineffective therapeutic targets in the first line.

## 2. CRC Biology

Colon cancer results from the aberrant proliferation of cells in polyps that gradually accumulate genetic alterations. Polyps consist of clusters of abnormal cells residing on the inside lining of the colon or rectum that may evolve to be non-neoplastic or neoplastic. As they transform, neoplastic polyps extend into the lumen of the colon and later infiltrate neighboring lymph nodes and distant organs. Some polyps are adenomatous, exhibiting low dysplasia. Adenoma is a non-malignant precancerous condition. It is already known that genetic events such as Adenomatous polyposis coli (APC) mutations, leading to continuous Wnt signal transduction, are capable of leading to this transformation [5]. The progression from adenoma to carcinoma is a complex process that is orchestrated by genetic alterations. Important early genetic events are microsatellite instability (MSI) and chromosomal instability (CIN) [6]. MSI occurs when repetitive small DNA sequences called microsatellites gradually accumulate mutations, suggesting that mismatch repair (MMR) proteins are not working effectively. CIN is the set of chromosomal abnormalities involving the abnormal segregation of chromosomes during mitosis, such as deletion amplifications and translocations [6]. Therefore, it is important to develop tools that can detect these early alterations in order to closely monitor the patient before the adenoma reaches a level of high dysplasia and eventually progresses to carcinoma. Early diagnosis of CRC appears to affect the outcome, as better treatment responses are observed at the initial stages of the disease. Consequently, prognosis is positively affected as well [7].

### 2.1. Liquid Biopsy–General Features

**Figure 1:**
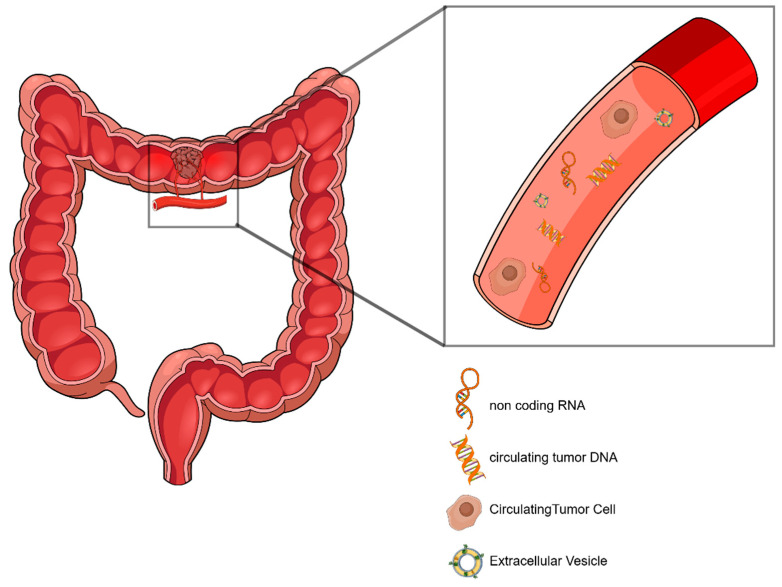
Circulating biomarkers in colorectal cancer. Circulating tumor DNA (ctDNA), circulating tumor cells (CTCs), extracellular vesicles (EVs), and non-coding RNA (ncRNA) are the main liquid biopsy representatives that are shed from the primary tumor and the metastatic sites into the bloodstream. CTCs are introduced into the circulation by intravasation and contain ctDNA and ncRNA. Both ctDNA and ncRNA can also be shed into the circulation via EV secretion from the tumor or by cell-death processes like apoptosis and necrosis. Similarly, EVs can either be actively shed into the bloodstream, serving as signaling-molecule carriers, or be products of apoptosis. (Figure created in the Mind the Graph platform, www.mindthegraph.com) (URL accessed on 7 October 2024).

Liquid biopsy is a set of minimally invasive techniques for the detection of biomarkers circulating in bodily fluids. The primary focus of liquid biopsy is the detection of cancer-specific biomarkers that are shed by the primary tumor and/or the metastases in the peripheral blood. Some representative target markers of liquid biopsy in the circulation include CTCs, circulating nucleic acids, and EVs (Figure 1) [8]. The aim of liquid biopsy is to utilize methods of analysis to enrich or isolate these markers based on their distinct phenotypic or physicochemical characteristics for their separation. In this way, it exploits the processes of metastasis carried out through the shedding of cells, vesicles, and biomolecules from the primary tumor and metastatic lesions, with the aim of extracting information that provides a holistic picture of the biology of the disease as well as the tumor’s heterogeneity and evolution. These goals are achieved because liquid biopsy techniques are minimally invasive (blood draws), or non-invasive (urine/stool sampling), in contrast to tissue biopsy. In addition to this, they can be performed frequently, and, thus, they allow for repeated measures for monitoring [9].

Liquid biopsy is a very promising approach that aims to increase the time points of sampling to monitor the disease in real time and to be a complementary method of diagnosis, treatment stratification, and prognosis. This is achieved owing to the fact that liquid biopsy is minimally invasive. However, most methods of isolation and analysis of circulating biomarkers still need standardization to be safely implemented in the clinic, and several of them are still high cost and do not provide satisfactory throughput. Furthermore, sample sizes need to be increased, and large prospective studies need to be conducted in order for liquid biopsy to be implemented in the clinical practice [10].

### 2.2. Circulating Tumor Cells–Biology and Clinical Utility in Early-Stage CRC

CTCs are neoplastic cells that are released into the circulation during the process of dissemination and are capable of initiating metastasis [11]. They effectively evade immune surveillance and often form aggregates with white blood cells or other CTCs [12]. The CTC-associated metastatic mechanism, as clinically described, consists of five stages: (1) The detachment of the cell from the tissue of the primary tumor. (2) The dissemination of the cell into the extracellular matrix through interactions with neighboring cells. (3) The intravasation and introduction of the tumor cell into the circulation. (4) The extravasation into a different organ tissue. (5) The promotion of invasion, colonization, and proliferation in the new environment through immunosuppressive interactions of the cancer cell with immune cells and neighboring normal cells [13]. These interactions are orchestrated by the genomic and molecular alterations that the CTC has accumulated, and thus, can transduce signals promoting its survival and proliferation, even in harsh environments [13].

A proportion of the solid tumor cancer cells, including CRCs, that are about to enter the circulation show the hallmark of phenotypic plasticity [14]. Particularly, they undergo a process of transdifferentiation called Epithelial–Mesenchymal Transition (EMT), in which the expression of epithelial proteins on the cell surface is reduced and the expression of mesenchymal proteins is induced, resulting in the cell gaining motility and intravasating easily into the bloodstream [15]. The opposite process, Mesenchymal–Epithelial Transition (MET), occurs during the establishment of the circulating cell in a distant organ [15]. For the non-targeted detection and analysis of CTCs of all phenotypes, enrichment techniques based on the physicochemical traits of CTCs have been developed. The Parsortix system (Angle plc) separates CTCs from the total amount of cells of the periphery, based on size and deformability, by trapping the larger CTCs in a cartridge [16]. However, size-dependent enrichment techniques are usually performed in cancer types with significantly larger cells compared with normal PBMCs, such as non-small-cell lung cancer (NSCLC) [16].

Furthermore, cancer cells appear to exhibit increased levels of glycolysis compared with their normal counterparts, which preferentially use the oxidative phosphorylation pathway for glucose metabolism in the presence of oxygen (metabolic reprogramming) [14]. The result of this preferred anaerobic process is the secretion of lactic acid into the cell’s environment and the removal of Na+, K+, and H+ ions from the cell’s surface, concentrating a negative charge around the cell membrane [17]. Taking advantage of this hallmark, Zhou et. al developed a CTC charge-based isolation method that uses positively charged magnetic beads to isolate CTCs, exploiting the electrostatic interactions between the positively charged beads and the negatively charged CTCs [17]. This marker-free approach serves to overcome the obstacle of detecting only cells that express epithelial markers, thus receiving a more informative “snapshot” of CTC heterogeneity by also isolating cells that have undergone EMT that would have been lost by an antibody-based technique [18].

**Figure 2:**
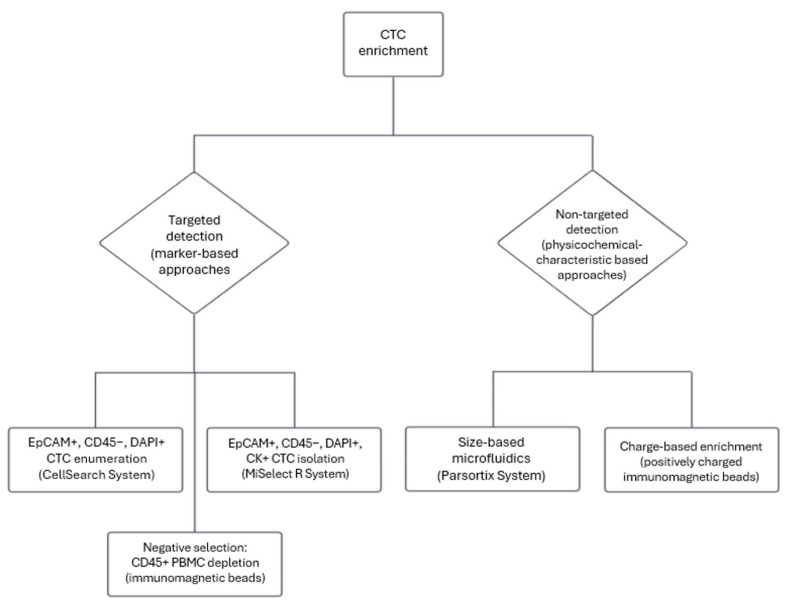
CTC detection flowchart. CTCs can be enriched from the rest of the peripheral blood components with an increasing variety of methods. This flowchart shows some representative techniques, classified into two categories: the targeted approaches that are based on phenotypic differences of CTCs from peripheral blood mononuclear cells, and the non-targeted approaches that utilize the distinct physicochemical characteristics of CTCs for their isolation.

The prognostic value of CTCs has been validated for patients with metastatic CRC (mCRC). Extensive studies on assessing the prognosis of patients with mCRC has led to FDA approval for the Cell Search System [19]. The Cell Search System (Menarini Silicon Biosystems) enriches and enumerates CTCs from a relatively small volume of peripheral blood (7.5 mL) and uses positive selection of cells bearing epithelial markers on their surface by immunomagnetic separation as the principal method (Figure 2). Ferrofluid with magnetic beads coupled with specific antibodies for epithelial marker molecules, such as Epithelial Cell Adhesion Molecule (EpCAM), is used. Any contamination by white blood cells is discarded after the use of dye-labeled antiCD45 antibody. CD45 is a marker expressed on the membrane of all white blood cells. Finally, nuclei are stained with 4,6-diamidino-2-phenylindole (DAPI) stain, and cell populations with CTC characteristics (epithelial markers on the surface, absence of CD45, presence of nuclei) are isolated and enumerated [20]. It has also been shown that the presence of CTCs in the blood of patients with early-stage CRC has prognostic value. In particular, van Dalum et. al observed that CTC detection in nonmetastatic patients prior to surgical removal is significantly associated with an increased risk of disease recurrence and CRC-related death [21].

Moreover, it has been observed that there is prognostic value in detecting CTCs in nonmetastatic CRC patients 2 to 4 years after surgery. CTC detection in this study was performed with Cell Search by increasing the blood volume analyzed from 7.5 to 30 mL, as CTCs in the early stages of the disease are extremely rare [21]. It has also been shown that CTC detection in postoperative patients with stage ΙΙ CRC is an independent prognostic marker that detects disease recurrence 4 months earlier than clinically established carcinoembryonic antigen (CEA) [22]. Yu et al. detected CTC in patient blood samples using negative selection by leukocyte depletion with antiCD45-coated, antibody-coupled magnetic beads. The residual CTC fraction was characterized using immunofluorescence in situ hybridization (imFISH) using DAPI for nuclear staining, CD45 for residual WBC exclusion, and Chromosome Enumeration Probes for hyperdiploidy detection [23]. Both groups stressed the predictive potential of CTC analysis, as they were led to the belief that CTC detection postoperatively is also considered a good candidate marker for the classification of patients for adjuvant therapy. A recent study by Lin et al. used the new MiSelect R System platform to isolate, count, and extract information relevant to the CTC phenotype from patients with early CRC. Specifically, MiSelect uses a microfluidics channel for enrichment, an optical detection system for enumeration, and immunofluorescence imaging to extract information from CTCs from peripheral blood samples. The study confirms the prognostic and predictive value of CTCs in risk stratification and in categorization for adjuvant therapy, as the presence of ≥1 CTC was significantly associated with worse Cancer-Specific Survival (CSS) [24].

Summing up, CTCs are tumor cells that enter the circulation and are capable of initiating metastasis in distant organs. CTCs are shed into the peripheral blood and evade the immune system by undergoing alterations in their phenotype and metabolism. The distinct phenotypic traits of CTCs serve in the development of enrichment techniques that aim to isolate CTCs from the rest of the PBMCs and provide further characterization and analysis. As is shown in Figure 2, CTC enrichment methods can be divided in two major branches, those that exploit specific phenotypic characteristics and those that utilize a marker-free approach in order to capture a broader set of cell subpopulations, thus tackling the issue that CTCs show high phenotypic heterogeneity. Additionally, several studies have already highlighted the prognostic significance of the analysis of CTCs both in mCRC and in nonmetastatic CRC 2 to 4 years after surgery [19,21]. Furthermore, stratification of patients for ACT seems to be significantly assisted by CTC detection [24].

### 2.3. ctDNA Biology and Analysis in CRC

Cell-free DNA (cfDNA) is derived from cells that have undergone apoptosis or necrosis. In the case of a cancer patient, a small fraction of the total cfDNA comes from cells of the primary tumor or metastatic sites, and thus, is called ctDNA. Leon et. al were the first to observe an increased concentration of circulating DNA in the serum of cancer patients in 1977 [25]. Circulating DNA usually consists of DNA fragments 120–220 bp long, a length capable of carrying valuable information about the biology of the disease. Regarding the biology of DNA shedding, it has been suggested that the ability of CRC tumors to shed DNA is related to their size and the proliferative capacity of their cells rather than a specific mutational pattern [26]. The analysis of ctDNA is a fundamental element of liquid biopsy and provides several promising new biomarkers that can be used to obtain information relevant to prognosis, personalized therapeutic approaches, and real-time monitoring of disease progression. The analysis of ctDNA holds great promise for improving the clinical management of cancer, as ctDNA can carry point mutations; alterations such as insertions, deletions and MSI; as well as epigenetic modifications such as DNA methylation [27,28]. All the abovementioned serve as a pool of potential biomarkers that may shed light on cancer biology and management [28,29].

The methylation of gene primers is a process of silencing through transcriptional inhibition [27]. Neuropeptide Y (NPY) expression is observed to be lower in patients who have developed liver metastasis (CRC-LM), which is consistent with the observation that NPY-ergic neurons are reduced in CRC-LM cases compared with healthy livers [30]. Following these observations, NPY–gene methylation is a promising early biomarker of recurrence. It also serves as an early suggestion for reorientation of the received therapy, as hypermethylated-NPY ctDNA (meth-NPY) is found significantly increased before radiological progression [31]. Analysis of NPY–gene promoter methylation can be detected by methylation-specific Droplet Digital PCR (dd-PCR) in ctDNA from peripheral blood [32]. The SDC2 gene encodes the syndecan-2 protein. One of the key functions of this protein is to interact with proteins in the extracellular matrix and cytoskeleton, and therefore, altered expression of the SDC2 gene affects migration and proliferation [33]. Septin-9 is the protein encoded by the SEPT9 gene. Its important contribution to cytokinesis, vesicle trafficking, migration, and apoptosis make the SEPT9 gene a strong candidate for research on carcinogenesis and disease progression [34]. Methylation-driven silencing of SEPT9 and SDC2 has been studied extensively in the context of biomarker discovery for the early diagnosis of CRC. A meta-analysis by Wang et al., which used data from 12 studies with a total of 1574 patients and 1945 healthy individuals, concluded that SDC2 methylation analysis could be considered a potential new biomarker for CRC screening (pooled sensitivity of 0.81 and specificity of 0.95) [35]. Additionally, another meta-analysis comprising 1913 CRC patients and 2851 healthy individuals has shown that SEPT9 and SDC2 methylation analysis in plasma or fecal samples have similar sensitivity and specificity in the diagnosis of CRC, thus suggesting that further studies on epigenetic markers are indeed required [36].

In addition to the detection of mutations and modifications in ctDNA, the presence or absence of ctDNA at various time points of the disease as well as ctDNA clearance can be a useful biomarker. The analysis of ctDNA and the extraction of conclusions from the resulting data can be performed with tumor-informed or tumor-agnostic approaches. In the first case, which is the most common, ctDNA is analyzed with prior knowledge of the genomic landscape of the tumor. In contrast, in the more recently implemented tumor-agnostic methods, the tumor is not analyzed beforehand, so that the process is faster and more cost-effective. Chan et al. showed that tumor-informed ctDNA analysis outperforms the tumor-agnostic approach in terms of both analytical sensitivity and the sensitivity of detecting recurrence. The same group also supports that longitudinal ctDNA analysis is a safer approach than a “landmark” analysis in predicting and detecting recurrence [37].

The analysis of the CIRCULATE-Japan GALAXY observational study used a tumor-informed ctDNA testing approach in which up to 16 tumor-specific single nucleotide variations were detected in tumor samples and then in cfDNA [38]. The prognostic value of the presence of ctDNA in the window of minimal residual disease (MRD) in patients with early CRC after surgery was demonstrated. In addition, ctDNA clearance helps with understand whether treatment efficacy is achieved in patients receiving adjuvant chemotherapy (ACT). More specifically, ctDNA positivity is associated with both an increased risk of recurrence and a more unfavorable overall survival (OS). Furthermore, MRD-positive patients had a significant benefit from receiving ACT. Post-operative ctDNA clearance was associated with favorable DFS and OS, and therefore, its importance in evaluating treatment efficacy in patients receiving ACT was highlighted [38]. The importance of ctDNA detection in blood samples of early CRC patients after surgery was also demonstrated by Kasi et al., as the interim analysis of the BESPOKE study supports the prognostic significance of ctDNA-based MRD positivity as an indicator of disease recurrence and also as an indicator of benefit from ACT [39]. The choice of adjuvant therapy in early-stage CRC is a matter of debate. The importance of ctDNA detection in early CRC was assessed by the DYNAMIC clinical trial. Here, 302 patients were assigned to ctDNA-guided management and 153 to standard management. It was shown that not administering adjuvant therapy to ctDNA-negative patients with stage II disease did not worsen the recurrence-free survival compared with patients receiving therapy. Therefore, the results of the DYNAMIC study suggest that liquid biopsy may be an informative tool for adjuvant therapy selection in patients with early-stage CRC [40].

The above observation highlights that treatment de-escalation in patients at high risk for adverse events who exhibit ctDNA negativity is of equally high importance. This is because it is crucial to remember that chemotherapy side effects are a significant drawback resulting from a non-personalized approach to disease management. However, for the de-escalation decision based on ctDNA detection to be robust, it is necessary to develop techniques of as high sensitivity as possible to minimize the risk of false negative results. Also, a longitudinal ctDNA assessment in these patients seems to be a safer approach, ideally combined with clinicopathological characteristics [41].

### 2.4. Non-Coding RNAs as Liquid Biopsy Biomarkers—The Role of EVs in ncRNA Analysis

RNA molecules that do not originate from the transcription of protein-coding genes are commonly called non-coding RNAs (ncRNAs). A growing body of studies have focused their attention on investigating the regulatory roles of ncRNA molecules in tumorigenesis and progression (such as transcription inhibition or their function as oncogenes or tumor suppressors), as it is observed that ncRNA molecules are also dysregulated in many types of cancer, including CRC [42]. The most commonly studied ncRNA families are microRNA (miRNA), long non-coding RNA (lncRNA), and circular RNA (circRNA). In addition, ncRNAs are found enriched in biological fluids such as plasma and blood serum, making them attractive potential biomarkers of liquid biopsy approaches [42].

EVs are membrane-limited vesicles secreted by cells that enclose a variety of cellular components. Their isolation is widely performed in liquid biopsy, as they help to keep the enclosed molecules stable, especially if the molecules are sensitive to hydrolysis or enzymatic cleavage, such as RNA [43]. Exosomes form a subset of EVs of endosomal origin that are 60–140 nm in diameter [44]. They have been identified as a means of intercellular communication, through which tumorigenesis and metastasis can be promoted [45]. To identify exosomes, Transmission Electron Microscopy (TEM) is one of the technologies used. However, the difficulty in distinguishing exosomes from EVs due to the limitations in separation methods has led to the suggestion of the term small EVs (sEVs) to be implemented instead, according to MISEV guidelines [46]. Thus, EVs offer an additional tool of liquid biopsy in the analysis of circulating biomarkers such as ncRNAs [47,48].

It has been reported that the exosome-specific miRNA, miR 17-92a, and the lncRNA, CRNDE-h, are interesting potential biomarkers for early diagnosis and treatment monitoring in early CRC [49]. NcRNAs further carry the interest of therapeutic targeting. A recent study shows that miRNA-372-5p promotes immunosuppression. MiRNA-372-5p expression takes place in CRC cells and causes the enhancement of Programmed Death Ligand-1 (PDL-1) expression in Tumor-Associated Macrophages (TAMs), resulting in a reduction in CD8+ T cells and immune evasion. Future investigation is therefore considered important in order to assess miRNA-372-5p as a prognostic marker as well as a therapeutic target [50]. Interestingly, it has also been shown that ncRNA molecules have the potential to provide a diagnostic insight at the early stages of the disease. A study by Pan et al. shows that hsa-circ-0004771 is found at high concentrations in the serum of patients with early CRC, in contrast with tumor tissue samples of the same patients. It is therefore indicated that the detection of hsa-circ-0004771 may help in the early diagnosis of CRC [51]. Finally, the assessment of treatment performance is of great importance for decision making by clinicians. H19 lncRNA appears to promote resistance to Fu-5, a commonly used chemotherapeutic agent. The proposed mechanism of chemoresistance involves binding of H19 to the downstream SIRT1 gene, thus counteracting the action of Fu-5. Therefore, ncRNAs such as H19 may serve as both markers for selecting a therapeutic approach and therapeutic targets [52].

EVs also enclose proteins, metabolites, and other analytes of interest for liquid biopsy approaches. These biomarkers have been shown to effectively mirror their parental cancer cells. Sensitive analysis methods such as mass spectrometry-based proteomics and metabolomics can give a comprehensive insight into the proteomic and metabolomic profile of EVs, respectively [53]. However, the extraction of information from the proteins detected in EVs regarding diagnosis or treatment stratification should still be interpreted with caution, as it has been shown that EVs show significant enrichment in specific pathway proteins compared with their cancer cells of origin [54]. More specifically, key proteins, including KRAS, mTOR, PDPK1, and MAPK1 were found enriched in sEVs derived from three CRC cell lines by Heck et al. It is known that the aforementioned proteins play a very important role in epithelial carcinogenesis.

EVs and exosomes are also actively evaluated in the clinical research area. The detection of exosomes and the analysis of exosomal biomarkers appear to be of value in the early diagnosis of CRC. A study by Zheng et al. shows that exosomal circLPAR1 appears to be significantly increased in plasma exosomes of CRC patients compared with those of healthy donors and patients with other cancer types, showing high specificity, and thus, it may serve as an early diagnosis biomarker for CRC [55]. Another study by Zhao et al. showed that EVs showing increased expression of miR-181a-5p contribute to the modification of the extracellular matrix to promote colorectal–liver metastasis (CRLM). It also appears that miR-181a-5p is a promising potential new biomarker of poor outcomes for CRLM patients [56]. Further studies to overcome the separation and isolation obstacles of EV subtypes are, thus, required to shed light on CRC-related pathways.

### 2.5. Tumor-Educated Platelets—A Liquid Biopsy Perspective of the Tumor Microenvironment

Platelets actively interact with tumor cells as a component of the tumor microenvironment, resulting in alterations in gene expression, and thus, in their phenotypic profile. The so-called tumor-educated platelets (TEPs) circulate and contribute significantly to tumor growth and dissemination [57]. TEPs have recently been demonstrated as a potential liquid biopsy source for early cancer diagnosis and for cancer treatment monitoring [58,59]. By modifying the so-called megakaryocyte-derived transcripts and proteins, platelets respond to tumor-derived molecular signals and interact by activating RNA profile alterations in the tumor cell’s microenvironment [60,61,62]. The techniques for TEP analysis involve RNA sequencing and reverse transcription–polymerase chain reaction (RT-PCR) replication of non-coding and ribosomal RNA from platelets derived from cancer patients’ plasma. Fu et al., 2021, prospectively investigated TEPS in patients with CRC who received adjuvant chemotherapy [63]. Blood samples were obtained, and 15 different gene mutations involved in cancer invasion, such as MYC, IL33, PTGS2, PTGER2, and VEGFB, were analyzed before and after surgery, during pre- and postoperative chemotherapy, as well as during the follow-up period [63]. The role of the platelet-to-lymphocyte ratio (PLR) was also investigated, as the patients were subgrouped according to the PLR levels. In the primary and secondary outcomes, the subgroup analysis showed that the chemotherapy patients with thrombocytosis and increased PLR level > 130 had a longer overall survival in comparison with patients who did not receive chemotherapy, implying that PRL should work as a factor to determine the group of patients whose tumors’ microenvironments are chemo-sensitive and will probably benefit from chemotherapy (HR: 0.371, 95% CI: 0.212–0.649) [63]. Also, the group of patients who showed a reduction in the platelet-to-lymphocyte ratio in the different time points of liquid biopsy analysis during the chemotherapy treatment had a lower recurrence rate compared with patients with thrombocytosis and high PLR, but the results were not statistically significant (*p*: 0.13) [63].

Similarly, Yang et al., 2022 investigated the tendency of PPR (postoperative-to-preoperative platelets ratio) in a retrospective cohort of patients who underwent surgery for colorectal cancer in Taizhou First People’s Hospital. To avoid possible interpretation limitations, patients who had received adjuvant therapy or who had other health conditions such as autoimmune diseases that could affect the platelets’ physiology were excluded. They showed that in both the development and the validation cohort, high TEPs and PPRs were associated with poor postoperative prognosis, functioning as independent prognostic factors both in the univariate and multivariate analysis. The predictive ability of PPR according to the time-dependent ROC curve was counted at 0.702 and 0.602 for 1 year and 5 years post-operatively, a fact that was enhanced when the PPR and TEPs joint index was applied in the analysis [64].

Further induction of TEPs into everyday clinical practice includes the creation of large databases with mutational gene expressions from TEPs analyzed from liquid biopsies in large case series with colorectal cancer, like PltDB, which can be used to separate patents with colorectal cancer from those with non-malignant diseases [65].

### 2.6. Prognostic Utility of Liquid Biopsies

The predictive and prognostic value of liquid biopsy markers in the setting of CRC has been recently highlighted in the literature [66,67]. An increasing number of cancer centers have registered several studies regarding CTCs to investigate the ability to predict the recurrence rate following surgery. The first large, randomized study in patients with adenocarcinoma was conducted by Tol et al., 2018, where blood samples from CAIRO2 study participants were used for CTC assessment [68]. The median PFS was statistically significantly different in patients with high baseline CTCs compared with low baseline CTCs (13.7 versus 22 months, respectively, *p* < 0.0001; HR 2.2 (95% CI 1.7–2.9)) [68]. The correlation between CT imaging and CTC levels was also determined [68]. A statistically significant concordance was shown between patients who showed a radiologic clinical response and the patients with low baseline CTC (*p* = 0.034) [68]. However, the CTC method was unable to detect patients with progressive disease (PD), as only 17% of patients with PD had simultaneously high levels of CTCs, resulting in a low positive predictive value [68]. In the multivariate analysis, CTC was shown to be the strongest early predictor for PFS both at baseline and at 1–2 weeks [68].

A large number of trials investigating the use of liquid biopsies in evaluating the prognosis of patients with CRC have been registered in the ClinicalTrials.gov database (Table 1). Tan et al., 2018 presented the results of a systematic review and meta-analysis, with 15 articles and a total of 3129 patients, where OS and PFS were investigated among CTC-positive and CTC-negative individuals [69]. Patients with detectable CTCs had worse overall survival and progression-free survival rates expressed in HR values (2.36, 95% CI 1.87–2.97, *p*: 0.006 and 1.83, 95% CI 1.42–2.36, respectively) [69]. However, different protocols of blood sampling and detection procedures, and inconsistent cut-off values, in each study added great heterogeneity sources in the meta-analysis [69]. The subgroup analysis revealed that the prognostic value of CTCs was statistically significant in studies with follow-up longer than 24 months, in the same way that specific detection time intervals and detection methodologies did not affect the statistically significant effect [69].

In the post-surgical setting with curative intent, Reinert et al. published, in *JAMA Oncology*, the results from a multicenter prospective study in Denmark with 130 patients with stage I to III CRC, who received surgery during the time period between 2014 and 2017 [70]. Circulating DNA was analyzed from 795 blood samples pre- and postoperatively (122 and 673 samples, respectively) and then quantified and sequenced with NGS sequencing [70]. The ctDNA sample was considered positive when more than two somatic mutations were detected. Patients with ctDNA-positive samples already at postoperative day 30 were shown to have a significantly higher recurrence rate (70% vs. 11.9%). Also, statistically significantly reduced recurrence-free survival (RFS) was correlated with positive ctDNA samples (HR: 7.2; 95% CI, 2.7–19.0, *p* < 0.001) [70]. This correlation remained statistically significant in the multivariate analysis that included also the disease stage and lymphovascular invasion as, impressively, ctDNA was found to be the only significant prognostic factor [70].

The same study achieved the highlighting of the predictive value of circulating DNA, which can indicate the subgroup of patients that continuously have residual disease and probably will not benefit from systemic chemotherapy [70]. More precisely, among 58 patients with available blood samples for post-adjuvant chemotherapy, 100% (7/7) of the patients with positive circulating DNA relapsed, in comparison with only 7 out of 51 (13.7%) patients with negative ctDNA who relapsed, after systemic therapy [70]. In the multivariate analysis, ctDNA remained the only significant factor for recurrence after adjuvant treatment. No association was found between baseline ctDNA and CEA status and clinical outcome. Patients with positive ctDNA and elevated CEA pre-operatively had a significant worse OS compared with patients with both biomarkers negative or CEA positive but ctDNA undetectable (*p* = 0.014) [70].

In the neoadjuvant setting, the effect of pre-operative circulating DNA was investigated in a substudy of the GEMCAD 1402 phase II randomized trial with patients with locally advanced rectal cancer (LARC) who received either neoadjuvant chemotherapy, with six cycles of aflibercept plus mFOLFOX6 (Arm A), or single mFOLFOX6. Blood samples for ctDNA were obtained from 72 patients from both arms (a total of 144 paired samples), and finally, 62 samples were adequate for sequencing and data analysis [71]. A total of 23 patients (66%) showed a complete ctDNA wash-out post neoadjuvant treatment, whereas only 4 patients (11%) had a persistently positive ctDNA both at baseline and after systemic therapy [71]. Furthermore, 20% of the patients had undetectable ctDNA from the baseline to the time point before surgery [71]. Measurable ctDNA before surgery was correlated with a statistically significantly increased risk of recurrence and reduced overall survival in comparison with cases with undetectable ctDNA (HR: 4.029; 95% CI, 1.004–16.16; *p* = 0.033 and HR: 23.95% CI, 2.4–212; *p* < 0.0001, respectively) [71].

Also in the metastatic disease, researchers from the MD Anderson Texas lately presented the results of a cohort of 63 patients with colorectal metastasis who underwent metastasectomy from 2016 to 2018 [72]. Liquid biopsies were received postoperatively and at specific time points during follow-up from the patients who had achieved a potentially curative hepatectomy [72]. Seventy somatic mutations related to carcinogenesis were able to be detected with next-generation sequence analysis from the segregated ctDNA [72]. The presence of a positive liquid biopsy (i.e., at least one detected mutation) postoperatively was statistically significantly associated with worse two-year overall survival compared with patients with negative liquid biopsies (70% vs. 100%, *p* = 0.005) [72]. More interestingly, the detection of more than four mutations along with positive resection margins were the factors that were significantly correlated with a worse prognosis (2-year OS: 41%, *p* < 0.001) [72]. This can be attributed to the onset of heterogenous clonal subpopulations resistant to systemic therapy. A positive correlation was also demonstrated between radiologic evidence of metastatic disease and positive liquid biopsies (77% of CT with metastatic disease had positive liquid biopsy), but not with serum CEA [72].

### 2.7. Prediction of Resistance to Treatment and Real-Time Monitoring of Response to Therapy

Bartak et al., 2022 proposed a protocol with serial blood collections from 55 patients (total of 367 plasma samples) with both nonmetastatic (32 nmCRC) and metastatic (23 mCRC) CRC to map the alterations in tumor molecular identity during systemic chemotherapy [73]. The levels of circulating DNA as well as the modifications in the level of methylation of the promoter SFRP2 and SDC2 genes were evaluated at different time points from the start until the end of the study [73]. The cut-off of >10% elevation or reduction in the cfDNA levels was set as considerable to detect a clinically significant alteration. Both in the nonmetastatic and the metastatic CRC disease, a decreased cfDNA concentration was observed in the majority of patients who remained in complete remission (67% of the nmCRC, *p* < 0.05) and stable disease (100% of mCRC patients, *p* < 0.05) [73]. The ROC curve analysis of the cell-free DNA as an indicator of tumor progression or remission showed a sensitivity of 83% and a specificity of 94% (AUC = 0.956, 95% CI 0.906–1.000, *p* < 0.0001) [67]. Furthermore, 28 samples were used for the evaluation of the SFRP2 and SDC2 methylation levels. Patients with progressive disease in both the mCRC and the nmCRC were shown to have significantly higher amounts of methylated SFRP2 and SDC2 allele copies in comparison with those with stable or total remission disease [73]. It is worth mentioning that the methylated allele frequencies for those two epitopes were not different between the patient groups at the start of chemotherapy [73].

The study from Reinert et al. aimed to monitor the response of 10 patients with positive postoperative ctDNA samples to adjuvant chemotherapy by obtaining consecutive blood samples during and after treatment [70]. The patients that did not manage to negate the circulating DNA in blood samples under adjuvant chemotherapy (4 (50%) out of 8 patients with available samples) had a disease recurrence during the follow-up, indicating that the value of ctDNA possibly reflects the systemic therapy’s lack of capacity to eradicate the residual disease [70,74].

In the metastatic setting of CRC, ctDNA has been proven to sufficiently monitor the molecular clones that emerge during the course of treatment, especially in the RAS, BRAF, and EGFR status assessment, to consider the re-challenge with anti-EGFR treatment [74,75]. Martinelli et al. 2021 presented in *JAMA Oncology* the results from the comparison of cetuximab plus avelumab to rechallenge therapy with anti-EGFR agents in patients with initially RAS wild-type (WT) mCRC [76]. Patients with *RAS/BRAF* WT ctDNA had mOS of 17.3 months (95% CI, 12.5–22.0 months) compared with 10.4 months (95% CI, 7.2–13.6 months) in patients with mutated ctDNA (HR, 0.49; 95% CI, 0.27–0.90; *p*  =  0.02) [76]. Sartore-Bianchi et al. presented the results of the CHRONOS trial that tested the re-challenging of anti-ΕGFR antibodies in mCRC [77]. The backbone of the trial lies in the fact that upon failure with anti-EGFR antibodies, patients with RAS and BRAF wild-type, metastatic CRC and no target-based mutation can undergo re-challenging with anti-EGFR therapy. This approach resulted in a response rate of 8–20% [77]. However, ctDNA liquid biopsy monitoring had never been used in a prospective, interventional way to map the mutational status of the cellular clones [77]. A total of 52 patients were screened with ctDNA analysis; 36 were RAS/RAF/EGFR wild-type and 27 were able to be enrolled for panitumumab (anti-EGFR) rechallenge [78]. Stable disease was achieved in 11 out of 27 (40%, 95% CI: 24–59%) patients and lasted more than 4 months in 9 out of 11 patients (82%) [78]. The implementation of liquid biopsies in the screening of patients who will potentially benefit from anti-EGFR rechallenge offers the potential for tailored patient management with limited available therapeutic options [78].

### 2.8. Comparison Between Liquid and Tissue Biopsies and Upcoming Limitations

A fundamental issue in determining the clinical utility of liquid biopsies in managing CRLM is establishing the concordance rate with tissue biopsies from primary and metastatic sites [79]. A multicenter study in France evaluated the RAS mutations in paired tissue samples and liquid biopsies from chemotherapy-naïve patients with metastatic CRC between July 2015 and December 2016. Of these patients, 80% (329/412) had conclusive ctDNA results, with a concordance κ coefficient between plasma and tissue RAS status at 0.89 (95% CI: 0.84–0.94) and accuracy at 94.8% (95%, 91.9% to 97.0%) [80]. Deviations were observed in a total of 17 patients, of whom 3 patients had RAS plasma mutations but no RAS tumor mutations, while 14 patients had RAS tumor mutations but no RAS plasma mutations [80]. Primary tumor resection, metachronous metastases, the absence of liver metastases, and peritoneal carcinomatosis were significantly more frequent among the 83 patients with inconclusive ctDNA results. The factors that were statistically significantly associated with inconclusive ctDNA results included primary tumor resection, metachronous metastases, the absence of liver metastases, and peritoneal carcinomatosis [80].

Siena et al. reported results from a prospective analysis in patients with mCRC, confirming that serial plasma biopsies are more inclusive than tissue biopsies for evaluating *RAS*-acquired mutations under the selective pressure of anti-EGFR treatment [81]. In 15 patients with paired tumor tissue biopsy and plasma samples, BEAMing on LB showed a far higher *RAS* MT emergence rate than tissue analysis by BEAMing and NGS (57.1% vs. 7.1% and 9.5%, respectively, *p* = 0.008), though without an impact on PFS [81]. However, only the RAS mutations were investigated by liquid biopsy in this study, leaving aside other known molecular mechanisms of disease resistance [81].

Similar discordances were observed in the NGS analysis of RAS mutations in available tumor tissue and plasma samples in a subgroup of 92 patients of the CAPRI-GOIM trial with first-line FOLFIRI plus cetuximab [82]. RAS mutations were detected in 33 of the 92 patients, both in tumor tissue and plasma samples. However, 10 patients were found to be RAS-tissue-mutant and wild-type in plasma, whereas 10 cases were identified as wild-type in tissue biopsy and mutant in liquid biopsy, with a rate of concordance to 78.3%. When ddPCR was used in the plasma samples, RAS mutations were detected in two additional cases that had been found as wild-type [82]. However, RAS mutations were detected in all 10 wild-type cases in tissue biopsies when ddPCR was applied. Median overall survival was similar according to the RAS mutational status between tissue and liquid biopsy tissues, suggesting that liquid biopsies are at least an equal tool to select for mCRC patients who might benefit from anti-EGFR monoclonal Abs [82].

## 3. Limitations and Conclusions

The implementation of liquid biopsies to gain complementary information regarding tumor evolution and heterogeneity is of great potential. However, the exact applications in which liquid biopsies can be used remain unclear. The increasing CTC count as well as the concentration of ctDNA during a round of chemotherapy that can be detected after treatment (i.e., no response or disease progression) may add to the stress experienced by CRLM patients with exhausted therapeutic options. More standardization is required for ctDNA and CTC tests, as there are not enough unanimously acknowledged assays for usage in clinical settings. Furthermore, many different assays have been employed in various research projects, and up to now, more head-to-head comparisons of assays that are commercially accessible need to be performed. Additionally, data indicate that renal function affects cfDNA clearance, a necessary aspect to be considered when interpreting ctDNA studies among patients with active cancer and concomitant chronic renal dysfunction.

Challenges regarding the design and conduct of clinical trials using ctDNA and CTC have emerged with the effort of measuring and monitoring the micro-metastatic disease when the macroscopic disease cannot be identified radiologically in the adjuvant treatment setting [83]. The rationale is to stratify those patients who will benefit from escalated adjuvant therapy and avoid toxicity in patients for whom intensified adjuvant treatment can be safely avoided [83]. ctDNA has been studied mostly as a surrogate endpoint to identify patients with a high risk of relapse because of the well-established detection techniques. By randomizing patients with a high-risk tumor profile, as defined by clinicopathological characteristics, and those with undetectable serial ctDNA to groups of either observation or adjuvant chemotherapy like in the VEGA-trial protocol, it is possible to identify the patients who will benefit from sparing overtreatment [84]. However, in the review by Verschoor et al. of trials investigating ctDNA-guided adjuvant treatment of solid tumors, it is commented that often, ctDNA-positive patients are not prospectively randomized into two interventional arms, but rather, randomization occurred in retrospective post-hoc analysis [70]. As those techniques have a high conduct cost, it is inevitable that a cost-effectiveness analysis should be added in the trials’ design [85]. Additionally, while liquid biopsies have been gaining in use among clinicians, most patients with high-risk clinicopathological features will still receive chemotherapy even if they present with undetectable biomarkers [85]. The optimal study design to address this issue has been proposed by the DYNAMIC trial, where patients with CRC in the adjuvant setting were randomized into two arms: the ctDNA-guided and the clinical risk-guided arm. Those in the first group with negative ctDNA deferred adjuvant treatment with no impact on the anticipated RFS, as mentioned [40].

In practice, determining the clinical value of liquid biopsies requires the optimization of method sensitivity and specificity while accounting for the possibility of false positive findings owing to clonal hematopoiesis of uncertain potential [3]. In the case of CTCs, their extreme rarity in early cancer stages and their great variety in size, density, and electric charge still make the current identification methods debatable in efficacy [3]. Additionally, intra-tumor heterogeneity makes it challenging to develop assays able to sufficiently capture the total genotypic and phenotypic variations that circulating tumor biomarkers exhibit, such as CTCs that have undergone EMT or new tumor-specific methylation patterns and mutations [3]. Regarding exosomes, ultracentrifugation, which is currently one of the indicated methods, includes practical limitations concerning the aggregation of lipoproteins and protein aggregates along with EVs [86]. To overcome this, density-gradient techniques can be combined with ultracentrifugation; however, this restricts the possibility of distinguishing molecules with the same density as EVs [86].

For this reason, continuous new research studies on “exploiting” complex tumor-specific biology for the development of novel liquid biopsy assays is required.

In conclusion, liquid biopsies have created new opportunities for cancer diagnosis, such as enhanced prognostication; assessment of therapy response, early relapse, and MRD detection; and tumor development tracking in relation to cancer treatments. Early findings on liquid biopsy testing for common mutations present a desirable and economical alternative to the existing method of tissue-based DNA mutation analysis. Repeated tissue biopsies in this situation might be performed only in response to unclear or erroneous negative liquid biopsy findings. To further establish and describe the function of liquid biopsies in the treatment of patients with CRLM, large-scale prospective studies are endorsed.

## Figures and Tables

**Table 1:** Registered clinical trials in colorectal cancer investigating the utility of liquid biopsies for evaluating clinical outcomes.

Clinical Trial ID	Population	Clinical Outcome	Liquid Biopsy Method	Status	Estimated Number of Participants	Sponsor
NCT04852653 (RECC-EV)	Adenocarcinoma of the rectum	Response to neoadjuvant treatment by chemotherapy alone or by radiochemotherapy	Onco-exosomes and/or exo-DNA as a response to neoadjuvant treatment after 6 months follow up	Recruiting	40	University Hospital, Bordeaux, France
NCT06076811 (DANISH.MRD)	Stages I–III adenocarcinoma receiving intensive surgery	3-year disease-free survival	ctDNA	Recruiting	1600	University of Aarhus, Denmark
NCT06111105 (GUIDE.MRD-01-CRC)	Stage III adenocarcinoma with received curative intention surgery	The 3-year recurrence-free survival	ctDNA	Recruiting	440	Guardant Health, Inc.
NCT05935384 (SIBYL)	Stages III–IV colorectal, non-small-cell lung, and breast cancer	Disease progression, RECIST, PFS, Lead Time	ctDNA (Guardant 360)	Recruiting	440	Guardant Health, Inc.
NCT04776655 (LIBImAb)	RASmut at liquid biopsy and RASwt on tissue	Progression-free survival	ctDNA	Unknown Status	280	Azienda Unità Sanitaria Locale Reggio Emilia, Italy
NCT05031975 (ERASE-TMZ)	Stage III or T4N0 stage II colon cancer	The activity in terms of seroreversion of TEMIRI consolidation regimen administered to patients, 2 years disease free	ctDNA	Unknown Status	35	Fondazione IRCCS Istituto Nazionale dei Tumori, Milano, Italy
NCT04259944 (PEGASUS)	Stage III and T4N0 stage II colon cancer	Number of post-surgery and post-adjuvant false negative cases	ctDNA	Active, not recruiting	140	IFOM ETS-The AIRC Institute of Molecular Oncology, Milan, Italy
NCT03401957	Metastatic with cetuximab-based regimen as first-line setting	Time duration between the start of cetuximab treatment and detection of RAS mutation	ctDNA	Unknown status	120	National Health Research Institutes, Taiwan
NCT04258137 (COPE)	Locally advanced/unresectable and/or metastatic solid tumor	Overall survival	ctDNA	Recruiting	332	Institut Bergonié, Bordeuax, France
NCT04425239 (Phase II) (IMPROVE)	Metastatic colorectal, IMPROVE trial	Progression-free survival up to one year after randomization	cfDNA	Completed	151	National Cancer Institute, Naples, Italy
NCT05524012 (PRIMO)	Locally advanced rectal cancer stages II–III with neoadjuvant chemoradiotherapy (CRT)	Tumor regression grading (TRG)	CTC	Recruiting	50	Jena University Hospital, Germany
NCT04554836 (Phase II) (MoLiMoR)	MoLiMoR, stage IV adenocarcinoma with FOLFIRI-based first-line therapy with or without cetuximab	Progression-free survival	ctDNA	Active, not recruiting	144	TheraOp, Germany
NCT06509126 (Phase III) (IMPROVE-2)	Metastatic colorectal cancer panitumumab plus Folfiri (IMPROVE-2)	Time to treatment failure	ctDNA	Recruiting	500	National Cancer Institute, Naples, Italy
NCT03699410 (observational) (LIBReCa)	Locally advanced rectal cancer (T3 or T4 and/or N+) requiring long-course nCRT	Negative prognostic value	ctDNA	Terminated	25	Dimitri Christoforidis, Ente Ospedaliero Cantonale, Bellinzona, Italy
NCT03751176 (Phase II) (BEYOND)	Second-line treatment after first-line panitumumab and FOLFOX in wild-type mCRC	Progression-free survival (PFS), conversion rate of RAS/BRAF status after second-line treatment	ctDNA	Unknown Status	31	Grupo Espanol Multidisciplinario del Cancer Digestivo, Spain
NCT06287814 (observational) (FRENCH.MRD.CRC)	Stages I–III colon and rectal cancer, curative intent resection surgery	MRD assessment	ctDNA	Not yet recruiting	70	University Hospital, Montpellier, France
NCT06287723 (observational) (FRENCH.MRD.CRLM)	Stage IV liver metastasis	Three-year disease-free survival	ctDNA	Not yet recruiting	30	University Hospital, Montpellier, France
NCT04735900 (Interventional) (FOLICOLOR)	Metastatic colorectal cancer patients receiving first-line FOLFOX/FOLFIRI and panitumumab	Optimization of the cut-off value for NPY methylation in liquid biopsies	ctDNA	Unknown status	60	University Hospital, Antwerp, Belgium
NCT04232891 (observational) (REDCLOUD)	Adenocarcinoma of the colon or rectum with synchronous metastatic disease (localized in liver or in liver and lung) eligible for surgery immediately or after neo-adjuvant treatment	Relapse rate in MRD+ patients	cfDNA	Unknown status	141	Fondazione del Piemonte per l’Oncologia, Italy
NCT04776837	Metastatic colorectal, pancreatobiliary, or esophagogastric cancer	Treatment response at first scan	ctDNA	Completed	200	Massachusetts General Hospital, USA

## Data Availability

No new data were created or analyzed in this study.
